# Are Men Aging as Oaks and Women as Reeds? A Behavioral Hypothesis to Explain the Gender Paradox of French Centenarians 

**DOI:** 10.4061/2011/371039

**Published:** 2011-11-24

**Authors:** Frédéric Balard, Isabelle Beluche, Isabelle Romieu, Donald Craig Willcox, Jean-Marie Robine

**Affiliations:** ^1^Equipe Démographie et Santé, INSERM, Centre Val d'Aurelle, Parc Euromédecine, U710, 34 298 Montpellier Cedex 5, France; ^2^Fondation Nationale de Gérontologie, 49 rue Mirabeau, 75015 Paris, France; ^3^Department of Human Welfare, Okinawa International University, 2-6-1 Ginowan City, Okinawa 901-2701, Japan; ^4^INSERM, U988 Site CNRS, rue Guy Môquet, 94801 Villejuif Cedex, France

## Abstract

Since the 1990s, several studies involving French centenarians have shown a gender paradox in old age. Even if women are more numerous in old age and live longer than men, men are in better physical and cognitive health, are higher functioning, and have superior vision. If better health should lead to a longer life, why are men not living longer than women? This paper proposes a hypothesis based on the differences in the *generational habitus* between men and women who were born at the beginning of the 20th century. The concept of *generational habitus* combines the generation theory of Mannheim with the *habitus* concept of Bourdieu based on the observation that there exists a way of being, thinking, and doing for each generation. We hypothesized that this *habitus* still influences many gender-linked behaviours in old age. Men, as “oaks,” seem able to delay the afflictions of old age until a breaking point, while women, as “reeds,” seem able to survive despite an accumulation of health deficits.

## 1. Introduction

The title of this paper builds on the famous fable of Jean de la Fontaine (1621–1695) *The Oak and the Reed* in which he compares the two plants facing the natural elements (Table 2). The moral of the fable is that the oak remains immovable while the reed bends into the wind, but when the wind becomes stronger, the oak is uprooted while the flexible reed survives. This metaphor illustrates the hypothesis we develop to explain part of the gender paradox noted by Allard and Robine [1] as a result of a large national survey on centenarians: “*…women are more prevalent in old age but in poorer overall health. It leads to an impossible challenge: if you want to become a centenarian, be a woman, you're more likely to achieve the breakthrough but once you get there, be a man, as you will be in better shape.*” Comparing the responses of the reeds to the wind with the adaptation of elderly to the inherent frailty process that progresses with age, we defend the hypothesis that men, as oaks, have the capacity to cope with the challenges of old age until a breaking point, whereupon they die, while women, as reeds, are more flexible and can adapt and survive.

## 2. Materials and Methods

### 2.1. Data from Previous Epidemiological Studies on French Oldest Old

In order to illustrate our hypothesis, we put into perspective various observations related to the French oldest old population coming from three different studies: *In the Search of the Secret of Centenarians* (1990–2000) (A la recherche du secret des centenaires (SIECLE and Ipsen Foundation)), *European Challenge for Healthy Ageing* (ECHA, 2003-2004), and *GEnetics of Healthy Aging* (GEHA, 2004–2010). The observation of the gender paradox was made over a ten-year period by Allard and Robine based upon a study of 3500 centenarians alive in France in 1990. Indeed, the Ipsen survey (1990–2000) followed 800 centenarians, 95 men and 705 women, including Mme Jeanne Calment (a women!), the oldest person who ever existed and who lived until 122 years old. Women were more numerous than men in the centenarian population in 1990, and it is still the case today. 

The figures (Table 1) show that women in old age are more numerous than men, with a sex-ratio (i.e., number of women divided by the number of men) strongly increasing with age. Moreover, women live longer according to French National Institute of Statistics (INSEE): life expectancy at birth, in 2010, was 84.8 years for women and 78.1 for men (Bilan démographique 2010, Insee Première 1332—January 2011). The longevity gap was 6.7 years between men and women. Beyond the French context, when we look at the living supercentenarians (people having reached the age of 110 years) around the world in the Gerontology Research Group database (as of January 17, 2011 http://www.grg.org/Adams/E.HTM), we observe that there are 79 women for 4 men, a sex-ratio of 19.8. Therefore, women live longer and are more numerous than men, and this situation becomes more pronounced with increasing age. If health, that is, the absence of disease or good functionality, contributes to longevity, then logically old women should be in better health than old men, that is, have less disease and disability. On the contrary, Allard and Robine showed that “*in the three standard dimensions of functional abilities, physical (strength, mobility, agility), sensory (vision and hearing) and cognitive (memory, attention, mental performance, and affect), men aged over 100 years have better performance than women*.” It was one of the first observations of the gender disability gap.

First, let us summarize the results of the Ipsen survey, the first French national survey on centenarians. A thorough standardized clinical examination was performed by the general practitioner (GP) of the centenarians. According to this medical examination, 65.2% of men were evaluated as having a “good” or “very good” health status, while only 56.5% of women were this healthy while 10.7% of women were evaluated as having a “poor” or “very poor” health while only 3.4% of men were at this bottom end of the health spectrum. Regarding vision, 35.1% of men had “good” or “very good” vision, while only 27.19% of women were in a similar condition. Centenarians totally blind or with bad vision were 22.0% for women and 19.1% for men. Unlike the other data, the centenarians who had “good” or “very good” hearing were more numerous in the female population, 28.2% against 21.1% in the male population. In the Ipsen survey, the cognitive health of the centenarians was tested using the 10 questions of the Pfeiffer test. This test is a screening test, similar to the MMSE (though shorter) for possible cognitive impairments and dementia. Men who made no errors numbered 39.1% while women numbered only 12.9%.

Since this study, no comparable survey on centenarians has been run in France, but we can examine the French data of two European studies: *European Challenge for Healthy Aging* (ECHA) [2] and *GEnetics of Healthy Aging* (GEHA), regarding activity limitations and cognitive impairments. ECHA involved centenarians or near centenarians and included 55 French men and 77 women. The use of the Mini-Mental State Examination (MMSE) resulted in an average score of 17.6 for men and 10.7 for women. Regarding the Activities of Daily Living (ADL) [3], 30.1% of men displayed no activity limitation while women numbered only 11.8% in this case. Similarly, while only 20.4% of men had difficulties with 4 or more ADL activities (the ADL evaluated were feeding, dressing, transferring, toileting, and bathing), 48% of women showed difficulties. The GEHA participants are younger because this study involved nonagenarian siblings. The average age of the 201 French male participants was 92.7 years old, and the average age of the 461 French female participants was 93.4 years. The MMSE average scores were 23.7 for men and 21.8 for women. Regarding the ADL, men without difficulties reached 66.2%, while women numbered only 46.4%. Men having difficulties with 4 or more ADL activities numbered 9.5%, while 16.2% of women showed difficulties. Even if we do not have specific data for the centenarians, 754,000 people were affected by dementia in France in 2010, 72% of them were women and 28% men, according to the last estimations [4]. 

The data presented here on health differences between men and women could be discussed in terms of data collection methodology or representativeness of the samples; however, they are sufficient to illustrate our main point, that is, “women live longer than men and they are more numerous in old age but men are in better health.” There are numerous studies in many countries confirming the above observations, and there are many hypotheses to explain the two components of these observations as we will explain below.

The literature dealing with the longevity gender gap is considerable. Each scientific discipline has developed several hypotheses to explain the causes of this phenomenon. Facts come from descriptive sciences, demography, epidemiology, and other fields, and from both the social sciences and the biological sciences. They can be linked with the whole life course of the subjects: we know that men are more affected by violent death (war, murder, work-related, traffic accidents, suicide, etc.), and their behaviour patterns expose them to risk factors such as excessive alcohol intake, tobacco and other drug consumption, or other risk raking behaviour. Accordingly, the survival selection is stronger for men than for women. Many hypotheses come from biological sciences: of course, physiologies of men and women are different. Genetic, hormonal, and phenotypical differences, such as body size, are well known. Other hypotheses come from psychological and social sciences: behaviours, education, and social roles are obviously gender-related; social inequalities between men and women during childhood and work life could lead to differing social and medical support, and so forth [1].  Box 1 lists some of the proposed hypotheses suggesting the complexity of the explanations of the differentials in health and longevity between genders. 

Eventually the survival advantage for women results in a larger percentage of women reaching older ages. For all age groups, more women survive, but despite living longer, they display more comorbid conditions. The mechanisms reported in Box 1 could explain the higher rates of disability among women. However, it is also possible that, for any morbidity level, men remain more active, outwardly displaying less disability, ignoring pain, discomfort, and risk, and this behaviour pattern contributes to a shortening of their life. It is this last aspect of the gender gap that we aim to explore in this paper from an anthropological perspective. Although some reviews are available on the gender gap (as briefly referred to in Box 1), there are far fewer that attempt to disentangle the relative contribution of the various biological and social factors and even fewer that examine this issue for the oldest old. Our goal is not to dissect the utility of the above hypotheses, but to suggest another one, based upon gender-linked behaviour patterns of the oldest old, which can contribute to partially explain the gender paradox in healthy survival among the oldest old.

### 2.2. In-Depth and Semistructured Interviews of Oldest Old Informants

For this purpose, we used qualitative data from comprehensive [5] and semistructured interviews of centenarians held between 2003 and 2007. Balard [6] met more than 100 centenarians or near centenarians (people at least 95 years old) while undertaking doctoral dissertation research under the framework of the ECHA and GEHA studies. He led comprehensive talks, in order to collect alternative points of view on the centenarians' daily lives, to be compared with the pictures painted by the epidemiologic questionnaires. The method was inductive, close to “grounded theory” [7]. The comprehensive talks were used to “*explore and examine research participants' concerns*” [8]. In addition to these comprehensive talks, Balard performed in-depth interviews [9] with a group of 14 key informants who were followed for 4 years and were subjected to 5 to 10 in-depth interviews each. 

In this context, the word “informant” means a person able to “bear witness to his or her group, society, and culture” [10]. All of our informants were aged 95 or older when we met them. Complementary interviews were also performed with their nearest relatives or proxies. Some of the informants died during the fieldwork and were replaced by new informants. The key informants were chosen according to their ability to communicate and their comfort in interacting. Even if people with dementia were not *a priori* excluded, people in advanced stages of dementia were not interviewed for practical and ethical reasons. Most of the interviews were face-to-face talks to avoid interference between the elderly and his/her family circle. The interviews were as close as possible to informal talks, without using a real interview guide but trying to “*go with the flow*” [9] and to follow the recurring concerns evoked by the centenarians during the comprehensive talks. As Bourdieu [11] recommended, it is “*better to listen to than to question*.” Listening is vital to understand the meaning of a discourse [12]. In this view, the qualitative interviews should provide to the researcher an access to the mental representations of the interviewees, to understand “*the meaning of respondents' experiences and life worlds*” [13]. As Charmaz [8] put it: “*Qualitative interviewing provides an open-ended, in-depth exploration of an aspect of life about which the interviewee has substantial experience, often combined with considerable insight*.” In our research, the particularity of our key informants was to be one of the oldest old, that is, a centenarian (or near centenarian).

In this context, the main themes of the interviewees were their life stories, current daily lives, thoughts, representations of old age, sense of purpose in life (*raison d'être*), assessment of the quality of their lives, and strategies to cope with frailty. Our posture was consistent with the advice of Goffman [14] to “*Subjectize yourself*” (to enter the subjectivity of the speaker).

The goal was to translate the thoughts or, in Lapantine`s words, the “representations” of the speaker, obtained from interviews centred on the interviewee's conceptions, reasoning, and subjective logic [15]. Centenarians were considered as “*meaning makers*” [16]. To facilitate the words of the informants, the interviewer opted to introduce himself as a student who meets the elderly to learn from their experience of life. This introduction turned out to be highly facilitating because it produced a dissymmetrical relationship in which the informant was in a position of superiority. The elderly was positioned as a teacher, imparting knowledge to a novice, thus preventing him/her from doubting statements given or perceiving the interview as a test, which may cause anxiety. In accordance with qualitative research, all interviews with key informants were tape-recorded and transcribed verbatim. Fieldwork and interviews were stopped when the data saturation level was obtained. The coding and analysis of the interviews were based on the meaning node obtained by the categories given by the informants' discourses. As a result of which, the usual theoretical frameworks on quality of life (psychological indices, life satisfaction, subjective well-being, etc.) and successful aging based on health and autonomy were not used.

## 3. Results and Discussion

“*Someone once asked me what he had to do to get old. Do not do anything, it comes. Old age comes without being noticed*” (Mme Emilia, 98 years old). (for ethical reasons, the names of the informants have been changed.) “*Physicians, families and all the others know a lot of things thanks to the intelligence of their brain and sometimes thanks to the intelligence of their heart. But we, we live inside old age. We feel old age and feeling is much more than knowledge.*” (Words of Mme B, quoted by C. Memin, 2001).

The analysis of the interviews revealed that the definitions of aging (vieillissement) and old age (vieillesse), given by the oldest old, did not really differ between men and women. While avoiding a deep discussion of ethnolinguistic concepts, we nonetheless should clarify one point. In French, there are two words, “*vieux*” (old) and “*âgé*” (aged), which could be translated as “old person.” The oldest old, and probably most people accept to be designated as “*âgé*” but refuse the word “*vieux*” which seems to be a taboo for them. In their representation, “*âgé*” refers to the aging process while “*vieux*” refers to an identity. When they are talking about their experience, our key informants explained that they feel they are “*aging but they are not old*.” Concerning the aging process, they describe it by saying “*It started there shortly*.” They feel the aging process through different symptoms. 

The first and probably the most important one is the *ability to walk*. For the oldest old, walking is a kind of index of the aging process. They assess their degree of aging by their performances and ability to walk. So the consciousness of the beginning of the aging process appears when they feel the first difficulties walking. Thereafter, when they have to reduce the distance they used to walk, or when they have to sit down several times during their usual walk, they consider that they are further in their aging process. In their representations, the one who cannot walk is “*the old one*” (le vieux) that's why M. Pierre (98 years old) could say when talking about the other residents of his nursing home “*Here, things have changed, more and more old ones (vieux) entered, you know people in wheelchairs*.” The second symptom that the oldest old consider as a marker of their aging process is the *feeling of fatigue*. They explain fatigue is at first occasional and then appears when they do usual activities: “*I am tired when I walk”* (M. Léon, 99 years old). Then fatigue comes also in the absence of activity. The mere presence of many people around them, and worse if they are engaged in discussion with them, causes fatigue. Just before becoming old (vieux), the oldest old explain “*even by doing nothing, we feel tired*” (M. Georges, 96 years old). The third symptom the oldest old consider as a marker of the aging process is the *feeling of vulnerability* that they express by saying “*Now I have to be careful with everything*.” Depending on the informants, “*having to be careful*” could focus on weather: “*I have to be careful not to get cold. I never go out when it rains or it is windy*” (M. Léon). It could also refer to the fear of falling, “*I am careful when I walk… if I fell, it would be terrible*” (M. Louis, 100 years). Some of the informants express their vulnerability related to others: “*Now, I have to be careful, if someone, if somebody attacks me, I could not defend myself*” (Mme Marie, 96 years old). The fourth and last symptom that oldest old recognize as markers of their aging process could be summarized as the *feeling of weakening.* They talk about the decrease of their senses: “*Today, I have difficulties to see*”; “*I do not hear very well*.” They admit being always impaired by small health problems while they were not used to be so in the past.

Through these different symptoms, the oldest old admit they are aging but they defend themselves against being labelled old (vieux). The old one (le vieux) is not aging, he is at the end of the aging process. In their representations, the old one is a human being living under a death sentence. According to the representation of the oldest old, the old one (le vieux) is first and foremost the one who is “*useless*.” In accordance with the social values of his/her generation, the oldest old consider that the social utility and the sense of life of human beings are in work and family. M. Aimé, 95 years old said “*Now I am old (vieux), I am unfit, useless… I cannot even start my rotary tiller. It does not matter if I die*.” Close to social utility, another marker of old age for the oldest old is the feeling of “*not being listened to*” by their entourage. Mme Emilia (99 years old) said “*The old ones (vieux) are the ones who are no more listened to*.” For the oldest old, “*being listened to*” is a real mark of social utility because as Mme Anna (96 years old) says “*I am not 96 years old without reason*.” A lot of them consider that today their social role is to pass on their experience to young people. In this view, those who cannot pass on their experience are useless and old (vieux). We have already explained that the old one (le vieux) is the one who cannot walk, he/she is also “*the one who lost one's head*.” This idea is close to the concept of “social validity”. Those who are demented are like the old ones (le vieux), they are marginalized and socially disqualified. The last qualifier used by the informants to describe the old one is close to the concept of dependence but it is expressed differently by the oldest old. As Mme Anna said “*You are old when you are at the mercy of everyone*.” Everybody depends on other persons but being “*at the mercy of somebody*” means to be at risk to lose control of one's life, to be forced or manipulated.

In the representation of the aging process expressed by the oldest old and in their ideas of old age, we find many elements of a cross-cultural definition of age already put forward by anthropologists [17–21]. Health, safety, functioning (mobility, capacity to act), and individual productivity (cf. “productive if it creates societal value, whether or not it is reimbursed”) [22] are some elements that many societies include in their definitions of aging. Aging implies a frailty process, according to Linda Fried, “*a physiologic state of increased vulnerability to stressors that results from decrease of physiologic reserves, and even dysregulation, of multiple physiologic systems*” [23]. According to our observations, frailty is not only physiological. In old age, frailty is also social, cultural, and psychological, involving the sense of identity. Indeed, the oldest old explain they suffer from the lag between “*their time*” and the society of today. They also suffer from a break in their identity, a sense of “diminished self” or what Levi-Strauss called when he turned 90 years old “*the feeling of being a broken hologram* (Reconstitution of memories of Roger-Pol Droit published in *Le Monde *29/01/1999. Discourses of Claude Levi-Strauss pronounced on 25/01/1999 in Collège de France.)” The old anthropologist described his life as a dialogue between his “*real self and his virtual self*.” To apply this concept to our informants, the *real self *is the frail old man or woman they see in the mirror and feel when confronting the challenges that come with “aged bodies” while the *virtual self* is the representation they have of themselves, which still preserves a living idea of the whole and a continuity with the past (almost a timeless identity). It is in this context that we hypothesize that men and women are not equal in their ability to face the various symptoms and related challenges of old age and dimensions of frailty. We hypothesise that, while men try to fight against the stigma of old age until death, women try to find a way to accept it and continue to live on. Through these gender-specific adaptive strategies, the gap between the “real self” and “virtual self” (in Levi-Strauss terminology) is more easily bridged for most of the oldest old women of this generation when compared to the men. 

According to the concept of “*generational habitus*” suggested by Mauger [24] and built on from the work of Mannheim [25] and Bourdieu [11], we hypothesize that the differences in behaviour between men and women, even at the oldest ages, are coming from a “*generational mentality*,” that is, “the typical ways in which people think and their attitudes toward life.” This theoretical position extends the notion of “*social generation*” [26] (i.e., “people whose unity comes from a particular mentality”). The concept of “*generational habitus*” is close to the idea of the existence of contemporaneous connections between individuals. “The generation creates a rather narrow circle of individuals who despite the diversity of the other factors at play, are linked into a whole by the fact that they lived the same major events and changes during their receptivity period” [27]. Mauger used the concepts of “*mental tools*” [28] and “*habit-shaping force*” [29] to illustrate how people are influenced by their generational membership. Over the life course, the main receptivity period is during childhood, when people acquire the gender stereotypes of their culture. Of course, the way of thinking, being, and doing evolves over the whole life course, but these cultural landmarks regularly resurface, especially when identity, sense of self, and self-esteem are threatened, as in old age, when gradual or sudden decline in physical capacity, loss of control over one's environment, or other age-related challenges emerge.

In order to illustrate the influence of the “*generational habitus*” for the behaviour patterns of the oldest old, we can evoke the “community of thought” which exists among our informants. Indeed, it appears that our informants share many values despite their difference in social status (occupation, level of education, and income). For them, family and work are of utmost importance. They severely reject behaviours jeopardizing the family such as divorce and remarriage. They have a very different conception of care than their children do. For them, care is part of the traditional system of mutual help and supportive relationships that they have known in their village or neighbourhood. In this view, care should not be a professional task and/or something that is paid for, except for specific medical care services. They consider that it is the role of their nearby family or close relatives to help them with daily tasks and provide support in old age. They cannot accept that they should pay for cleaning and cooking whereas they have (or they think they have) previously helped others in the past. This “*generational habitus*” still influences them in their daily choices, values, and thinking. 

The analysis of the interviews showed a clear gap between the representation of the social role of men and women for our informants. The large majority of people born during the first two decades of the 20th century in France were “country folk” even if some of them spent their adulthood in towns. As showed by Segalen [30], this rural environment was characterised by an important distinction between men and women concerning social identity, social role, and production functions. These findings are close to the work of Guttman [31] who describes men as “breadwinners,” active, and dominant, and women as “homemakers” who provide emotional support and accept dependency as part of their social role, that is confined mainly to the domestic sphere. In this context, Segalen [30] showed that despite the appearance of male domination, there was mutual support, solidarity, complicity, and reciprocity between men and women. Lebra [32] referred to the exchange of such mutual obligations as *reciprocal dependency* where there exists a reciprocity or exchange of dependency. Others have recognized that despite the appearance of subjugation and economic dependency in relation to their husbands, women wield considerable power and status within the domestic sphere that accompanies their role monopoly as full time homemaker. Feminist anthropological approaches have analyzed these social exchanges within the context of a public and private dichotomy where men wield political power as the “public face” of society, while the private space is the female space. Ortner [33] elaborated on this theme when she published a landmark article likening *female to male as nature is to culture*. This theory dealt with the perception that men are the upholders of culture whereas women are more associated with nature. Women are seen as closer to nature in reference to three dimensions: (1) women's bodies are seen as more *natural* since they are more involved with childbirth and childrearing; (2) the social roles of women that intertwine with child rearing are viewed as closer to nature, specifically confining women to the domestic realm; (3) social perceptions of the female psyche portray woman as closer to nature.

Of course, the power, status, and roles of men and women vary between cultures and time periods. Moreover, men and women have multiple overlapping and contextualized identities based upon gender, age, ethnicity, class, regional, and other differences that evolve over the life course. Some authors like Guttman [31] describe an androgyny process. Following his theory, the traditional gender role is stronger during what he called “*the parental imperative*” (i.e., to ensure the survival of children and the handing down of the social values. Once the children have reached adulthood, the parents could express the “*other-gender*” side of their personality). This phenomenon seems to be due to the softening of the traditional gendered roles that occurs with the aging process. Men become less authoritarian and more sensitive, whereas women accept less to be dominated and become more powerful. Guttman [31] used the image of the warrior who became a peaceful chief, or that of the “virile older woman.” Many anthropologists have shown that during adulthood, and especially after menopause, women's social roles, status, and identity change. For example in Gabon, according to Bikoma [34], “The non-menstruating woman takes part in strictly masculine demonstrations. She is integrated into the guard and is part of the Wise.” Many works have described how women's status changes, often rising as they grow older [35, 36]. Thus, it appears that the gender differences in behaviour change over the life course, most often appearing as a softening of the traditional gender characteristics. The child of one of our informants confirms “*Today it is different but when I was a child, my father was terrifying*.” 

Does very old age cause an increase in the androgyny process observed in middle-life or is there a specific moment in the life course in which the traditional gender role reappears? We consider that the answer is significantly different between traditional societies and postindustrial ones, such as the France of today.

The finding of Singleton [20] that “In some societies, when you get older, you see better (the author refers to the mystical and magical abilities often attributed to the oldest old in traditional societies) and you are better seen” does not seem to hold in our postindustrial societies. Indeed, the image of very old age is quite negative in France. It conveys the ideas of illness, handicap, and senility. As we explained earlier, the oldest old worry about losing their identity as well as becoming “*an old person*” (*un vieux*). They suffer from the feeling of no longer being part of society. It is possible that, facing this unknown and stressful event that represents very old age, one important adaptational strategy consists in taking refuge in what they consider the most important things (i.e., what they value most) in their life and identity. Men try to show that they are still strong enough, helpful (useful), cognitively aware and intact, and able to make decisions for themselves, while women concentrate on issues such as being emotionally close to their family members, seeing them happy in life, and maintaining other important human relationships. Thus, it appears that the components of the social identity of women are less challenged by the frailty process, offering them more possibilities to adapt to the losses associated with the aging process when compared to men. 

### 3.1. Men as Oaks: Preserving Their Virtual Identity at All Costs

“*Frailty implies to be no longer able to be the same while feeling the necessity to be oneself*” [37] (Journée d'études internationales: L'âge et le pouvoir en question. Intégration et exclusion des personnes âgées dans les décisions publiques et privées, Paris, 10 et 11 Septembre 2007).

The interviews with the male informants reveal that preserving their “virtual self” is one of the leitmotives of their dialogues and a constancy of their behaviour. They give much weight to the past when making current assessments of self. Their self-identity is based on their past roles and statuses as worker, breadwinner, and head of family. They accumulate anecdotes on their competence as workers. Some of them presented themselves as a kind of “stakhanovist” (exceptionally diligent and productive). M. Aimé “*With my horse, I worked every day from sunrise to sunset, I worked for me and for the others too*.” M. Léon: “*When I was 86, I used to prune vines 5 or 6 hours a day.”* Physical strength and physical endurance are two important dimensions of the self-identity they make efforts to lay claim to. Others have insisted on their skill at work like M. Henri (96 years old) who explained that he made a piece out of wood to replace a part of a tank engine during World War II. Generally, they are keen to report anecdotes which highlight their productive skills. They did their best to work as long as possible even if they were officially retired. For them, it is vitally important to prove that they are still valid, competent, autonomous, and able. With an interlocutor, their self-presentation leans towards “self-staging” [14] or presenting themselves in the light of their ideal self-image. When they became unable to go on with their work, they tried to find activities in which they could continue to prove they were competent and useful like gardening, or repairing and improving things around the home. Within their family, the men of this generation are used to being in authority and to be listened to, even if these characteristics weaken over time and with age. During the interviews, they insisted on showing that their opinion is still listened to and respected by their children to whom they continue to give advice. To survive in old age, the men in our study seemed to choose to stay immovable. They did not like to deal with their new and vulnerable self-identity and refused to be compared to the old ones (les vieux). However, there comes a time when the gap between their “real self” and the image they have of themselves is too large to continue to self-stage. Thus, when walking becomes too difficult, when it is impossible to preserve a useful activity in conformity to their identity, when they have the feeling that they are losing their authority because they are not able to manage daily affairs, men at these oldest old ages express a kind of surrender. We have already quoted the sentence of M. Aimé: “*Now I am old (vieux), I am unfit, useless… I cannot even start my rotary tiller. It does not matter if I die*.” M. Léon, who was a very positive centenarian during the first interviews, confessed 3 months before his death: “*Now I get to the end… I am too old*.” The events that precipitate the surrender and probably the “*premature*” death of the oldest men are various and personal. Some of them could not endure the death of their wife. In addition to the emotional suffering, they appear helpless without the person devoted to take care of their daily personal needs. Others could not deal with the idea of being physically disabled. Usually oriented to outdoor activities, they found it extremely difficult to accept that they must now stay home. When they felt they were losing control over their environment, their self-sufficiency, and perhaps most importantly their continuity in self, the oldest men seem to prefer to give up and die rather than accept a state of disability and/or the idea of further gradual decline. To use the terminology of Evert et al. [38], men seem to have the ability to survive as “delayers.” Therefore, as long as they can delay significant physical limitations and severe disability, they have the will to continue to keep going and literally stay alive. The behaviour of oldest old men is highlighted by their efforts to remain physically and mentally strong and active. This may contribute to explain their better physical and cognitive functional health status, compared to women, but also their tendency to take more risks and their excess mortality. Because they are giving such significance to their functionality and physical performance, the oldest men make a great deal of effort to walk even if it is dangerous for them and this risk-taking behaviour may lead to falls and fatal events. M. Aimé's daughter explained that the neighbour found her father lying in his garden after a fall on three separate occasions.

Ultimately some of them may prefer to die. Two of our male informants explained that they thought about committing suicide; the first because he could not endure the thought of himself as degraded and useless, and the second because he was obliged to leave his home to enter a nursing home. Statistics confirm the tendency for men to commit suicide more often than women. For instance, at age 85 and over, the suicide mortality rate in France is 6.5 times higher for men than for women [39].

We saw that men can train themselves more than women to perform various physical tasks. We also wonder whether they are not tempted to exaggerate when reporting on their health status and functional abilities. Do they fool themselves when self-staging? Do they try to bias reality when reporting their self-perceived health or ADL abilities? Cognitive tests or physical tests, such as the hand grip test, are *a priori* objective tests. Such behaviour implies they might be on the razor's edge. They delay or seem to delay the old one (le vieux) stigma until a breaking point. This behaviour may partly explain why they are less numerous among older ages and why they live shorter lives. Possibly, the stronger selection effect that occurs for men reinforces their behaviour and the feeling that they are in better health. Compared to other men of their generation, they are the survivors, the chosen ones. This “relative appreciation” [40] may result in a self-appraisal that they were the most robust and/or able of their birth cohort, it may be that they literally could not see themselves otherwise.

### 3.2. Women as Reeds: “Let Live” to Survive

The oldest old women report very different behaviour compared to men. Their “*generational habitus*” also influences their social roles. Although playing their major social role within the domestic sphere, contrary to their husbands, they were never the ultimate authority, even in their own home. When they were children, they lived under their father's authority and then, as a spouse, under their husband's authority. Thus, to be in authority is not something cultural, it is not a habit. In this generation, many women have worked as housewives. Their family role was firstly “*to take care*” of others, housework, childrearing, and help their husband in his activity. In their life, “*taking care of others*” is what better illustrates their self identity. When they were children, they used to help their mother at home. Then, when they were mother and wife, they were responsible for the health and the blossoming of their children and husband. Most of them were also helping their husband in his work, taking care of several tasks. Later, these women were in charge of their old parents or step-parents. The representations attached to their identity were neither physical strength, nor being in authority, but the ability to listen to and understand the needs of others, to be a person full of maternal affection for others. Their “*working*” role was mainly oriented towards the household and associated tasks [30]. The activities linked to their social identity were very often cooking, doing the washing or other household tasks, and childrearing. Their ability in sewing was a gender-specific ability that was highly valued. M. Aimé's daughter explains “*the death of my mother raised the issue of my father being alone. I mean when she was alive, she was really a servant for him. She did cooking; she prepared his clothes for grooming… she did everything for him*.” The daughter of a couple of nonagenarians testified in a letter “*My mother is 90. She weighs only 37 kg. Her entire existence was conducted according to her husband. She has to prepare his medicine, read for him (very painful), especially to serve the meal for him*.” The daughter explains that her mother sacrificed herself for her husband. Referring to this point we notice that this could partly explain why the protective effect of marriage in France is only valid for men in this generation [41]. Mme Emilia (99 years old) confirms that in her generation the role of women was dedicated to support other family members: “*When I was a child, I helped my mother… I had to take care of my young brothers and sisters [ *…*] I don't want to live with my children because I know what taking care of an old person implies, I have done that for my father and my mother-in-law*.” 

According to Cool [59], we note that there is “continuity in women's core social role of housewife” for this generation and we agree that women seem to show an ability to adapt to discontinuity and/or conflicting roles throughout the life course. Therefore, women of this generation appear more flexible and adaptable than men. Indeed, if the collected material shows a general continuity in the social role of the women of our sample, it also shows that they had to face many discontinuities, upheavals and challenges over the life course. We hypothesize that their greater flexibility and ability to adapt is partially explained by their “generational *habitus*” shaping them as “good girl” and “exemplary spouse.” As Segalen [30] expressed it: “Authority to men and power to women.” Women have learned not to fight against their “environment” but to adapt to it and to find the best ways to live in it. When they grew old, women did not feel the need to fight against old age stigma as men did. Physical strength, being in authority, and an identity based on their ability to be the head *of the family are not female prerogatives for this generation.* We observed that women more easily accepted having difficulties with some activities, such as gardening, walking outside the house, and could more easily give up such activities. We also observed that women were not emotionally injured when their children made decisions for them. They explained that as an old person, the most important thing for them was to see their children and grandchildren. They appreciated it when they were still able to cook for them. As Mme Emilia, and many oldest-old women explained “*now, I let myself live*.” What she means is that she chose “*not to fight against old age*” but to “*live with it*.” Confronted by a number of losses, such as memory or mobility, Mme Germaine says “*I do not care about that today; it is not the most important… what really matters is to see my children and their families in good health*.” Women seem to have a high ability to accept not being able to fully control their life, and their body, their fate. When they fail in the MMSE test, they appear less uncomfortable with this than men do. They do not consider that their identity and pride are at stake. In conclusion, women seem to have a better ability to accept and live with the various symptoms of old age and frailty. Therefore, even if they accumulate health problems, chronic degenerative diseases, functional limitations, and activity restrictions, even if they are no longer performing as they once did, they can survive. Referring to Evert et al.'s [38] terminology, women seem to be closer to the “survivors.” Not focusing on their losses, they are able to continue to live without experiencing a large gap between their “virtual identity” and their “real identity.” This behaviour was reported by all six oldest old female informants in our study. We can also notice that, while the majority of the men in the French sample of the GEHA study did their best to be successful with the physical and cognitive tests that were proposed, women seemed not to consider them so important. It is possible that for the women in GEHA, the interaction and the presence of someone to communicate with was more important than the tests *per se* and could partially contribute to explaining why they were less successful in the tests when compared to men. 

The hypothesis developed here is close to *identity theory, *as proposed by Burke [60]. We insisted on the “*generational habitus*” that influences the gender behaviour in old age and that we observed in the current cohorts of the oldest old people. Being much less self-centred than men, women of these cohorts found a way to relativize their frailty and live with it, instead of ignoring or concealing it as men seemed to do. The main part of these gender-based behavioural or adaptational differences in regard to frailty and old age possibly came from the “*generational habitus*” which constructed the physiological reserve, psychological attitudes, ways of thinking, values, and social behaviour of the oldest old men and women in a very different manner. Men and women of these birth cohorts have grown up, lived, and grown old as completely different social beings. Their gender-specific behaviour in old age continues to influence their longevity and quality of life in different ways. 

However, looking at the current homogenization of male and female social roles and behaviours (especially health-related behaviors), we can postulate that the gender gaps observed today among centenarians may decrease over time. More recent cohorts of women have partially adopted risky behaviours, such as smoking or drinking alcohol, which were the prerogative of men in the past, and increasing participation in what was once considered exclusively male occupations (transport, military, law enforcement, etc.) may expose women to more violent deaths due to accidents, homicide, and/or suicide. Indeed, in line with decreasing polarization of gender roles, the size of women's life expectancy advantage over men has been steadily shrinking in most developed nations (some exceptions such as Japan exist) for the past few decades. Moreover, men in developed nations seem to be paying more attention to their health in recent years when compared to men of previous generations, with recent gains in male life expectancy (in comparison to female) appearing to be related more to an acceleration of progress for males, rather than a worsening of health situation for women [61]. Decreasing male deaths from cancer (especially lung) and cardiovascular disease are the major mortality-related contributors [61, 62]. In this context, the gender gap in longevity will likely continue to decrease in the future as more women take up smoking and other health risk behaviors. But will this new “*generational habitus*” change the gender differences for the oldest old? The 200,000 centenarians who will live in France in 2060, according to the latest population forecasts, will have a “*generational habitus*” constructed in the postwar period, shaped by social, cultural, technological, economic, and behavioural forces far different than that of today's centenarians. They will have a much higher level of medical literacy. But will the behaviour of men and women be more alike as they enter the ranks of the oldest old? Of course only time will tell. The gender gap in longevity and disability will likely remain in a state of flux for some time due to the continual shifts in the social, economic, and behavioural dynamics that determine health and longevity for both women and men.

## 4. Conclusions

French people, who were born at the beginning of the 20th century (i.e., before 1915), display a “*generational habitus*” which clearly juxtaposes men and women in terms of social identity, family role, and sense of purpose in life. This *habitus* still influences many gender-linked behaviours and adaptational strategies, even at the oldest ages. We suggest that these differences in behaviour favour functional health for men and longevity for women. The “*generational habitus*” influences the representation of the “*raison d'être”* for men and women. The male “*raison d'être*,” for these cohorts, is more self-centric and oak-like explaining why, when the gap between their real and virtual identity and self-image as the provider and pillar of their family and community becomes too wide and difficult to sustain, they find it nearly impossible to go on. On the contrary, the female “*raison d'être*,” for the same cohort, is based more on their status in relation to others, in particular as givers of care, even if it is reduced to their mere presence next to those they love. They are more flexible, adaptive, and reed-like; therefore, even if more severely disabled than men, women can continue to survive.

**Quote from Claude-Levi Strauss on the Occasion of His 90th Birthday**

(Note that he continued on until he reached 100 years old, a centenarian, as did most of the subjects in this study.)

“Montaigne dit que la vieillesse nous diminue chaque jour et nous entame de telle sorte que, quand la mort survient, elle n'emporte plus qu'un quart d'homme ou un demi-homme. Montaigne est mort à 59 ans, et ne pouvait avoir idée de l'extrême vieillesse où je me trouve aujourd'hui. Dans ce grand âge que je ne pensais pas atteindre et qui constitue une des plus curieuses surprises de mon existence, j'ai le sentiment d'être comme un hologramme brisé. Cet hologramme ne possède plus son unité entière et cependant, comme tout hologramme, chaque partie restante conserve une image et une représentation complète du tout. […]Ainsi y-a-t-il aujourd'hui pour moi un moi réel, qui n'est plus qu'un quart ou la moitié d'un homme, et un moi virtuel, qui conserve encore vive une idée du tout. Le moi virtuel dresse un projet de livre, commence d'en organiser les chapitres et dit au moi réel: “C'est à toi de continuer". Et le moi réel, qui ne peut plus, dit au moi virtuel: “C'est ton affaire. C'est toi seul qui vois la totalité." Ma vie se déroule à présent dans ce dialogue très étrange. […] Je vous suis très reconnaissant d'avoir, pour quelques instants, grâce à votre présence aujourd'hui et votre amitié, fait cesser ce dialogue en permettant un moment à ces deux moi de coïncider de nouveau. Je sais bien que le moi réel continue de fondre jusqu'à la dissolution ultime, mais je vous suis reconnaissant de m'avoir tendu la main, me donnant ainsi le sentiment, pour un instant, qu'il en est autrement.”

“Montaigne says that old age diminishes us so that, when death arrives, it claims only a quarter or a half a man. Montaigne died at fifty-nine and surely had no idea of the extreme old age, in which I find myself today. Having lived to a ripe old age which I never expected to attain, and which is one of the strangest surprises I have experienced, I feel like a shattered hologram. This hologram no longer possesses its entire unity, yet, as with any hologram, each surviving shard preserves an image and full representation of the whole.

So today for me there is a real self, which is but a quarter or half a man, and a virtual self, which preserves a living idea of the whole. The virtual self is planning a new book and beginning to organize its chapters, and to the real self it says, “Your job is to carry on.” But the real self, which cannot carry on, says to the virtual self, “That's your problem. Only you see the thing whole.” My life nowadays is defined by this very strange dialogue.

I am very grateful to you whose presence here today and whose friendship have for a short time silenced this dialogue and allowed these two selves to coincide again briefly. I know full well that the real self will continue to melt away until the moment of final dissolution, but I thank you for reaching out and for an instant giving me the sense that it might not be so.”

Claude Levi-Strauss speaking extemporaneously to friends assembled to celebrate his 90th birthday (January 1999). (Translated from the French by Arthur Goldhammer.) Source: http://buffleheadcabin.com/post/6614364005/.

## Figures and Tables

**Box 1:**
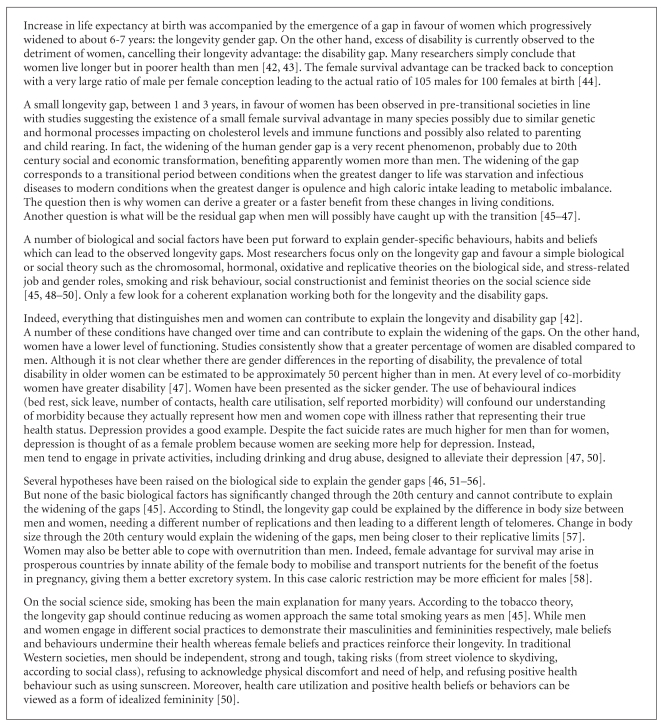
A sampling of hypotheses regarding the gender gap in health and longevity.

**Table 1:** Breakdown of the French population by sex and age, by January 1st 2011.

Birth cohort	Age	Total	Males	Females	Sex_ratio
1920	90	159965	45659	114306	2.5
1915	95	28413	5461	22952	4.2
1910	100	6926	694	6232	9.0

Source: INSEE, 2011.

**Table 2:** 

De la Fontaine, J (1668). *Les Fables choisies*.	Thomson, R (1806). *La Fontaine's fables*. Now translated from the French; Paris: Doyen.
Julaud, J-J. (2010). *Les Fables de la Fontaine* (new Ed), Paris: First Editions.	

Le Chêne et le Roseau (I, 22)	The Oak and Reed (I, 22)

Le Chêne un jour dit au Roseau: “Vous avez bien sujet d'accuser la Nature; Un Roitelet pour vous est un pesant fardeau. Le moindre vent, qui d'aventure Fait rider la face de l'eau, Vous oblige à baisser la tête: Cependant que mon front, au Caucase pareil, Non content d'arrêter les rayons du soleil, Brave l'effort de la tempête. Tout vous est Aquilon, tout me semble Zéphyr. Encor si vous naissiez à l'abri du feuillage Dont je couvre le voisinage, Vous n'auriez pas tant à souffrir: Je vous défendrais de l'orage; Mais vous naissez le plus souvent Sur les humides bords des Royaumes du vent. La nature envers vous me semble bien injuste. -Votre compassion, lui répondit l'Arbuste, Part d'un bon naturel; mais quittez ce souci. Les vents me sont moins qu'à vous redoutables. Je plie, et ne romps pas. Vous avez jusqu'ici Contre leurs coups épouvantables Résisté sans courber le dos; Mais attendons la fin. “Comme il disait ces mots, Du bout de l'horizon accourt avec furie Le plus terrible des enfants Que le Nord eût portés jusque-là dans ses flancs. L'Arbre tient bon; le Roseau plie. Le vent redouble ses efforts, Et fait si bien qu'il déracine Celui de qui la tête au Ciel était voisine Et dont les pieds touchaient à l'Empire des Morts	The oak one day addressed the reed, “Nature you may accuse indeed; A wren for you's a heavy load, The softest breeze that stirs abroad, That ruffles but the water's bed, Compels you to hang down your head; While I, like some proud mountain's brow, Not only stop the solar ray, But brave the blasts that round me play: Loud rowing storms to you, to me like zephyrs blow. Now, did you spring within the shade I throw, Were you beneath my sheltering foliage found, You would not suffer from the north unkind; I could defend you from the tempests round; But ye are seldom, save in marshy ground, Upon the borders of the realms of wind. Nature to you I really think unjust.” “Your pity,” answered him the reed, “I trust From goodness springs, but pray that pity spare; The winds that trees and mountains tear Alarm not me—unbroken still I bend. You hitherto, 'tic true, unshaken bear Their mighty blasts—but wait the end.” Just as he spoke, A tempest from the far horizon broke; Ne'er from the bowels of the north, Till then, came such a son of fury forth; The oak stood fast; the reed bowed down again. The winds then bursting with redoubled roar, Up by the roots the boasting giant tore, Whose cloud-capped head so proud did reign, Whose feet sank down to death's domain.
